# Safety and Efficacy of Early High Parenteral Lipid Supplementation in Preterm Infants: A Systematic Review and Meta-Analysis

**DOI:** 10.3390/nu13051535

**Published:** 2021-05-02

**Authors:** Kyunghoon Kim, Na Jin Kim, Sae Yun Kim

**Affiliations:** 1 Department of Pediatrics, College of Medicine, The Catholic University of Korea, Seoul 06591, Korea; journey237@catholic.ac.kr; 2 Medical Library, The Catholic University of Korea, Seoul 06591, Korea; kimnj@catholic.ac.kr

**Keywords:** lipids, preterm infants, growth, adverse effect

## Abstract

The objective of this systematic review and meta-analysis was to summarize the effects of early initiation and achievement of a high dose of parenteral lipids (≥1.5 g/kg/day reached within the first 24 h of birth) on growth and adverse outcomes in preterm infants. PubMed, EMBASE, and Cochrane databases were utilized to search for publications for this meta-analysis. Randomized controlled trials were eligible if data on growth or clinical outcome was available. The search returned nine studies. The mean proportion of postnatal weight loss (%) was lower (mean difference [MD]: −2.73; 95% confidence interval [CI]: −3.69, −1.78), and the mean head circumference near the term equivalent age (cm) was higher in the early high lipid treatment group (MD: 0.67; 95% CI: 0.25, 1.09). There was a favorable association of early high lipid administration with the incidence of extrauterine growth restriction (relative risk [RR]: 0.27; 95% CI: 0.15, 0.48). Generally, there were no differences in morbidities or adverse outcomes with early high lipid administration. Early initiation of parenteral lipids and high dose achieved within the first 24 h of life appear to be safe and endurable and offer benefits in terms of growth.

## 1. Introduction

The goal of nutrition for preterm infants is to mimic the intrauterine growth rate and to achieve favorable long-term developmental outcomes. Despite recent enhanced nutritional support in neonatal medicine [1,2], achieving this weight gain goal is still very difficult, especially during the first week of life when preterm infants are exposed to highest risk of undernutrition. Insufficient nutrition during this critical period results in cumulative energy and protein deficits that aggravate the postnatal weight loss [3].

Lipids are indispensable components for preterm infants in the early stage of postnatal life because of the essential fatty acid supply for growth and development and their high energy density. Recent meta-analyses provided evidence that the early initiation of lipids in preterm infants appears to be safe and tolerable but that there are no significant benefits of lipid administration before 2–5 days of age [4,5]. Additionally, a study in France reported that early introduction of parenteral lipids improved neonatal growth and that the lipid dose was positively associated with weight gain in very preterm infants [6] and possible later neurodevelopmental outcomes [7]. The latest recommendation suggested by the European Society for Pediatric Gastroenterology Hepatology and Nutrition (ESPGHAN) in 2018 showed that, in preterm infants, intravenous lipid emulsion (IVLE) can be started immediately after birth [8]. To date, “early” is not defined as an immediate time, but in a recently published randomized controlled study on the administration of IVLE in very preterm infants, researchers compared more immediate timing, within 6 h after birth versus between 12 and 24 h after birth, showing that the provision of a high amount of lipids early results in less weight loss and better head growth, in terms of the z-score [9].

In the aforementioned ESPGHAN guideline, for preterm infants, parenteral lipid intake should not exceed 4 g/kg/day because of adverse effects such as lipid intolerance; however, there was no consensus about how high a dose of IVLE was endurable and optimal in the early postnatal period. Additionally, there was little evaluation of long-term efficacy and safety, which makes concrete recommendations difficult to be established. Consequently, the objective of this meta-analysis is to find out the short-term efficacy and safety of early high IVLE introduction to preterm infants; dose of IVLE is equal to 1.5 g/kg/day or more within 24 h after birth. Anthropometric data and possible adverse effects associated with early high IVLE administration were investigated to determine the efficacy and safety.

## 2. Materials and Methods

### 2.1. Search Strategy

We performed a systematic review and meta-analysis in accordance with the preferred reporting items for systematic reviews and meta-analyses (PRISMA) guidelines [10]. We did a systematic search employing a protocol designed by an independent medical librarian (N.J. K) with three electronic databases: PubMed, EMBASE, and Cochrane Library. To qualify for inclusion, studies had to be randomized controlled trials (RCT) comparing interventions that differed only in the dose of parenteral lipids that were administered to preterm babies. We used the search terms listed in the supplemental file (S1) to search three electronic databases. We imposed no language or publication restrictions.

### 2.2. Study Selection

**Figure 1:**
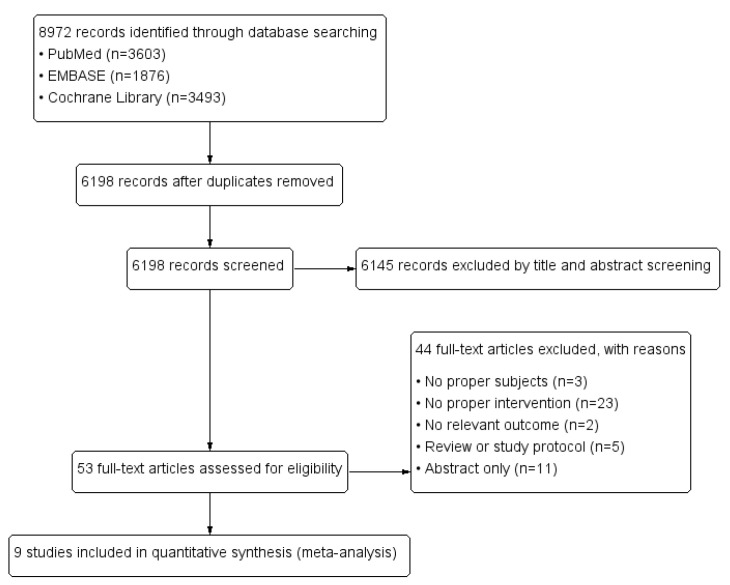
Overview of the selection process throughout the study.

Two reviewers (K.K. and S.Y.K.) independently evaluated the titles and abstracts obtained from the search for the first screening. After the initial exclusion process, two reviewers (K.K. and S.Y.K.) independently reviewed the full texts of the remaining articles to determine whether any articles met the eligibility criteria: an RCT design; a study group of preterm infants weighing <1500 g who were admitted to a neonatal intensive care unit (NICU), necessitating parenteral nutrition, and were administered any type of parenteral lipid emulsion within the first 24 h of life; and growth data included as an outcome measure. We resolved disagreement by discussion. We recorded the selection process in sufficient detail to complete the PRISMA flow diagram (Figure 1).

### 2.3. Data Extraction and Assessment of Risk of Bias

Both reviewers (K.K. and S.Y.K.) used a structured format to extract data from each eligible study. Data could be categorized as characteristics of the sample, intervention details, and measurement of outcomes. We resolved disagreement by discussion. Outcome measures were divided into primary outcomes on growth divided into six components and ten secondary outcomes. Primary outcomes were as follows: (1) weight gain rate (g/kg/day); (2) proportion of postnatal weight loss (%); (3) time to regain birth weight (BW) (d); (4) body weight near term equivalent age (TEA) (g); (5) head circumference near TEA (cm); and (6) extrauterine growth restriction (EUGR) defined by weight less than 10th percentile for postmenstrual age (PMA) based on the Fenton growth chart [11]. Secondary outcomes were as follows: (1) death before NICU discharge; (2) length of NICU stay (d); (3) incidence of bronchopulmonary dysplasia (BPD) or chronic lung disease (CLD) defined as oxygen therapy; (4) incidence of necrotizing enterocolitis (NEC) stage ≥2 on the modified Bell’s staging criteria [12]; (5) incidence of culture proven sepsis; (6) severe intraventricular hemorrhage (IVH) with a Papile classification of grade 3 or 4 [13]; (7) retinopathy of prematurity (ROP), defined as any stage of ROP after birth detected by ophthalmoscopy, as defined by the International Classification of Retinopathy of Prematurity [14]; (8) incidence of hypertriglyceridemia and serum triglyceride (TG) level (mg/dL); (9) incidence of hypoglycemia or hyperglycemia; and (10) serum total bilirubin (TB) level (mg/dL).

**Figure 2:**
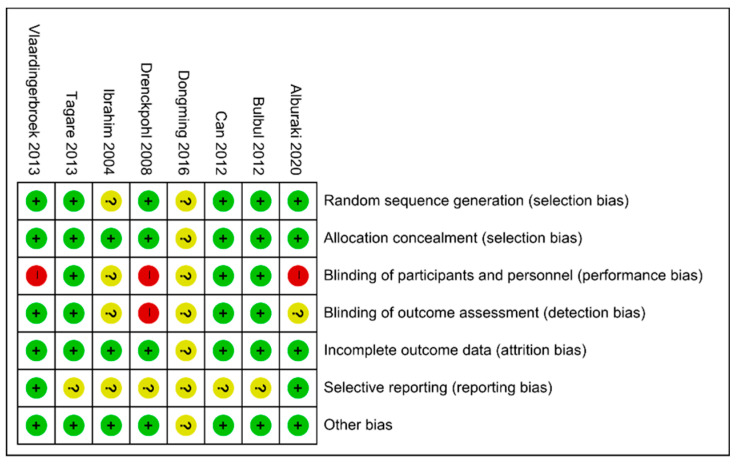
Risk of bias assessment of the randomized controlled trials included meta-analysis.

Two reviewers (K.K. and S.Y.K.) independently assessed the risk of bias for each study using the criteria outlined in the Cochrane Handbook for Systematic Reviews of Interventions [15] (Figure 2). We resolved disagreement by discussion.

### 2.4. Data Analysis

Review Manager 5.4 (The Cochrane Collaboration, London, UK) was used to perform analyses. To analyze the treatment effect and calculate a pooled mean of outcomes reported in more than two studies, the Mantel–Haenszel method was used for categorical outcomes, and the inverse variance method was used for continuous outcomes. All tests were two tailed, and a *p* value of less than 0.05 was deemed statistically significant. We used the *I*^2^ statistic to assess heterogeneity in the results of individual studies (*I*^2^ > 50% was used as a threshold indicating significant heterogeneity). We conducted sensitivity analyses when heterogeneity was distinguished. This was achieved by removing a study from the analysis to determine changes in *I*^2^ values and assess which studies play a significant role resulting in heterogeneity [16].

## 3. Results

### 3.1. Systematic Literature Search Results

A total of 8972 citations were initially screened in the databases, and 6198 individual publications were identified. Among these, 6145 studies were excluded after reviewing titles and abstracts, leaving 53 articles for full text review for eligibility (Figure 1). Finally, nine studies were selected with proper eligibility [9,17,18,19,20,21,22,23,24]. One study [20], which was a planned follow-up of a previous RCT [19], was based on the same population and was therefore considered an identical study. Therefore, eight publications were included in our final meta-analysis [9,17,18,19,21,22,23,24].

### 3.2. Sample Characteristics

Overall, 520 infants were included in our meta-analysis: 262 infants in the early high IVLE groups and 258 infants in the control groups. IVLE was initiated between 2 h and 24 h after birth. The starting dose was equal to 1.5 g/kg/day or more and was increased by 0.5–1.0 g/kg/day over 1–2 days to a maximum of 2.0–3.0 g/kg/day in the intervention groups, which means that the administration dose of IVLE reached equal to or more than 1.5 g/kg/day within 24 h after birth. On the other hand, IVLE was started between 12 h and 2 days after birth with a dose of 0.5–1.4 g/kg/day in the control groups, and the maximum doses were lower than 1.5 g/kg/day at 24 h after birth (Table 1). The risk of bias of the included studies was evaluated and is described in Figure 2. Follow-up was described in one RCT [19,20]. The characteristics of the included studies assessing the effect of early high IVLE (≥1.5 g/kg/day) versus control and early low (<1.5 g/kg/day) IVLE supplementation are presented in Table 2.

### 3.3. Primary Outcome Measures

**Figure 3:**
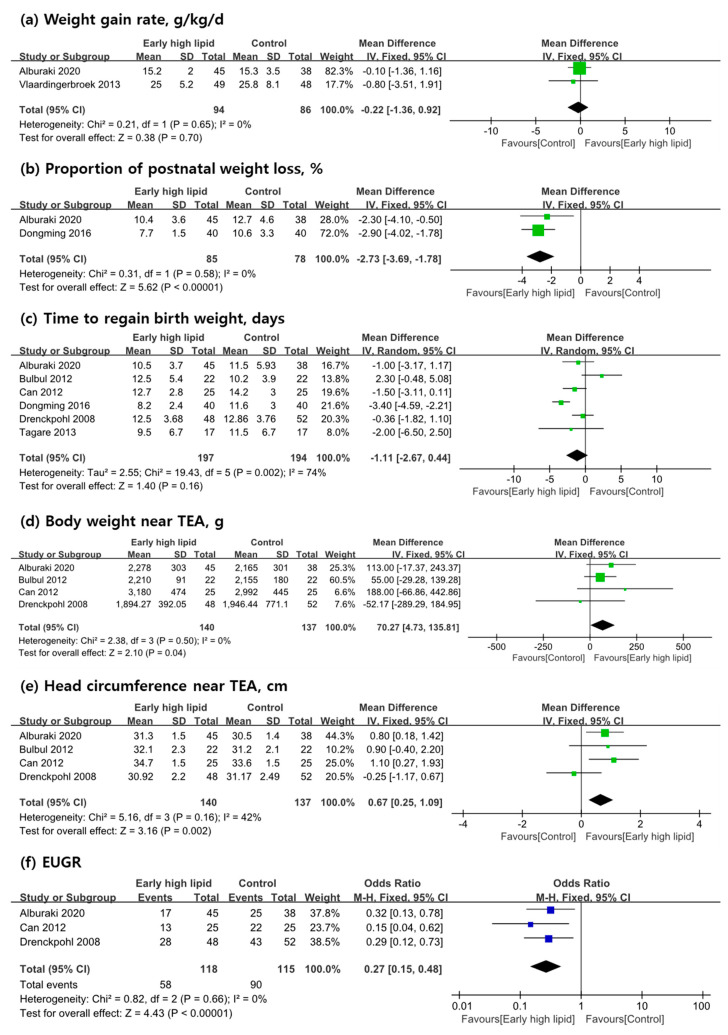
(**a**–**f**): Meta-analysis of the effects of early high IVLE that reached a lipid dose equal to or greater than 1.5 g/kg/day within the first 24 h on the growth of preterm infants compared with controls (random effects). IV, inverse variance; M-H, Mantel–Haenszel; CI, confidence interval; TEA, term equivalent age; EUGR, extrauterine growth restriction.

The weight gain rate was measured in two studies [9,19], We conducted a meta-analysis of these two studies, which showed no differences between the early high IVLE group and control groups (Figure 3a). However, there was a lack of consistency in the timing to measure weight. Alburaki et al. [9] reported mean weight gain from regaining birth weight to 36 weeks PMA, meanwhile the study of Vlaardingerbroek et al. [19], where growth until discharge home or until 40 weeks PMA was measured. Alburaki et al. [9] and Dongming et al. [17] documented the proportion of postnatal weight loss. A meta-analysis that included these two studies showed a significant favorable association of early high IVLE administration (mean difference [MD]: −2.73; 95% confidence interval [CI]: −3.69, −1.78; *p* < 0.00001; *n* = 163) (Figure 3b), but in two RCTs, the timeline of this outcome was inconsistent. Two studies were excluded, and the time to regain birth weight was reported in the remaining six RCTs [9,17,18,21,22,23]. A meta-analysis including these studies discerned no significant differences between the intervention group and control group (Figure 3c). As a consequence of considerably high heterogeneity with an *I*^2^ of 74%, sensitivity analyses with the removal of several far-reaching studies were conducted; however, the heterogeneity was not improved. Growth parameters until discharge home or TEA were measured in four studies. Alburaki et al. [9] reported growth anthropometrics measured at 36 weeks PMA; on the other hand, Can et al. [22] measured at 40 weeks PMA, and Bulbul et al. [21] and Drenckpohl et al. [23] checked the weight of the infant at discharge to the home. In aggregate, a meta-analysis of mean body weight near TEA that included four previous studies was performed [9,21,22,23]. The result showed a significant difference that favored the intervention group, which means that early high IVLE administration has a better effect on growth than the control parameter (MD: 70.27; 95% CI: 4.73, 135.81; *p* = 0.04; *n* = 277, Figure 3d). In the same four studies [9,21,22,23], the mean head circumference near TEA was measured and reported. A meta-analysis showed a significant difference in favor of the intervention group (MD: 0.67; 95% CI: 0.25, 1.09; *p* = 0.002; *n* = 277; Figure 3e). Three studies reported the incidence of EUGR [9,22,23]; Alburaki et al. evaluated the infants at 36 weeks of PMA, Can et al. at 40 weeks of PMA, and Drenckpohl et al. at discharge. A meta-analysis of these three studies was possible and showed an association between early high parenteral lipid administration and a lower incidence of EUGR (relative risk [RR]: 0.27; 95% CI: 0.15, 0.48; *p* < 0.00001; *n* = 233, Figure 3f).

### 3.4. Secondary Outcome Measures

In a subtotal of six studies [9,18,19,22,23,24], the overall mortality was 7.5% (15/201) in the early high IVLE group and 7.1% (14/198) in the control group. None of these studies reported a significant discrepancy in mortality between groups, and meta-analysis supported this finding. The length of NICU stay was reported in six RCTs [9,18,19,21,22,23], and no significant effect of early high lipid to admission length was disclosed.

**Figure 4:**
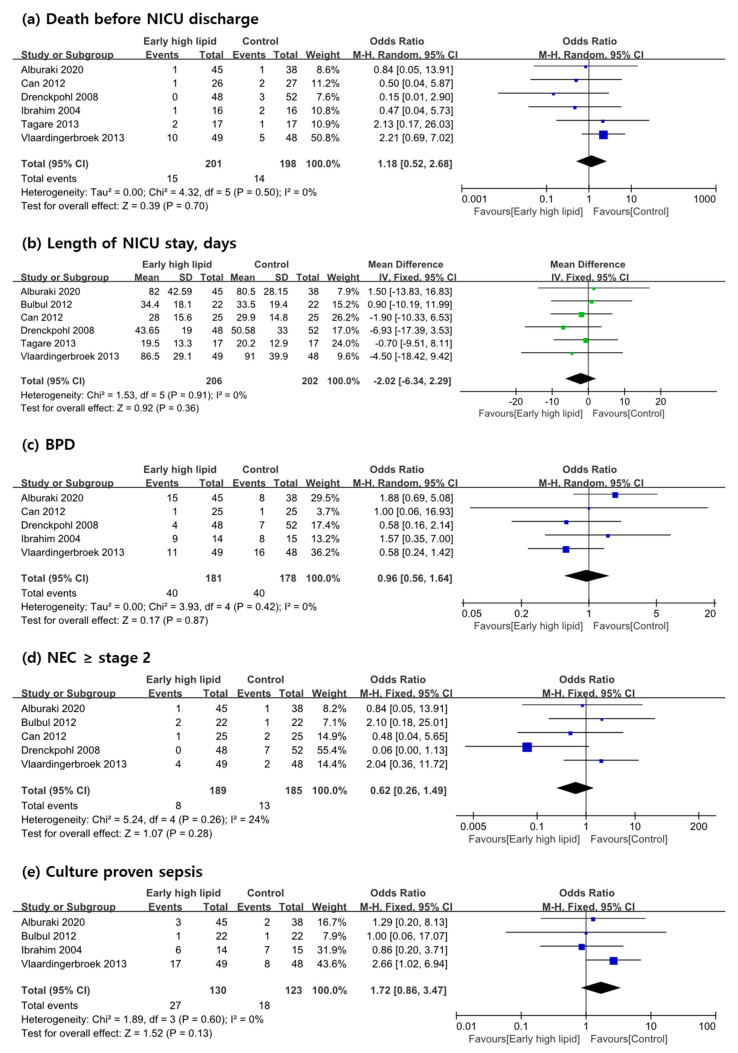

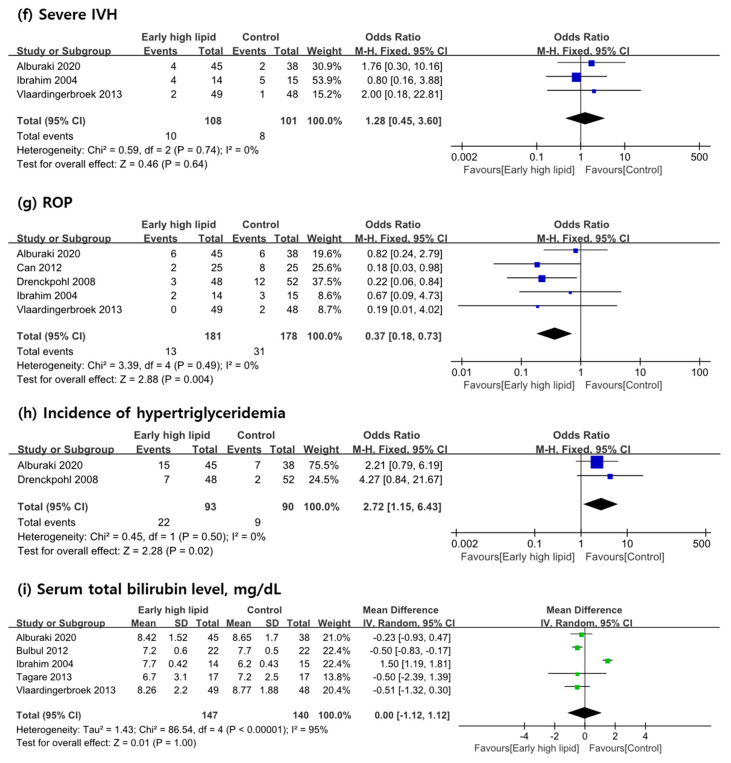
(**a**–**i**): Meta-analysis of the effects of early high IVLE that reached a lipid dose equal to or greater than 1.5 g/kg/day within the first 24 h on clinical outcomes and morbidities in preterm infants compared with controls (random effects). IV, inverse variance; M-H, Mantel–Haenszel; CI, confidence interval; NICU, neonatal intensive care unit; BPD, bronchopulmonary dysplasia; NEC, necrotizing enterocolitis; IVH, intraventricular hemorrhage; ROP, retinopathy of prematurity.

The incidence of BPD was reported in five studies. Alburaki et al. [9] diagnosed BPD on the basis of the National Institute of Child Health and Human Development workshop [25]. In the study of Vlaardingerbroek et al., this was diagnosed by physiologic definition with an oxygen reduction test [26]. In three studies by Can et al., Drenckpohl et al., and Ibrahim et al. [22,23,24], the incidence of requiring oxygen at a postmenstrual age of 36 weeks was described, and this could be used for the diagnosis of BPD or CLD. However, no significant effect of early high IVLE administration was confirmed in individual studies or in the current meta-analysis of these five studies (*p* = 0.87, Figure 4c). The incidence of NEC was described in five studies [9,19,21,22,23]. At the individual study level, only one study reported a significantly higher NEC incidence in the control group than in the experimental group [23]. The other four individual studies showed no significant difference, which was underscored by the meta-analysis of five studies (*p* = 0.28, Figure 4d). Alburaki et al. [9] reported early onset sepsis; Vlaardingerbroek et al. [19] reported late onset sepsis with culture proven; and Bulbul et al. [21] and Ibrahim et al. [24] defined sepsis as a positive blood culture not specifying the timing. None of the original studies reported a significant difference in the incidence of sepsis, and this was confirmed by meta-analysis of four studies (*p* = 0.13, Figure 4e). The incidence of severe IVH was reported in the studies of Alburaki et al., Vlaardingerbroek et al., and Ibrahim et al. [9,19,24]. Again, no significant effect of early high IVLE administration was confirmed in individual studies or in this meta-analysis (*p* = 0.64, Figure 4f). The incidence of ROP was described in five studies [9,19,22,23,24], although the applied definitions in each study were diverse. A meta-analysis of five studies showed an effect of early high IVLE administration on the occurrence of ROP, which favored intervention (RR: 0.37; 95% CI: 0.18, 0.73; *p* = 0.004; *n* = 359, Figure 4g).

Blood samples of infants included in each RCT were obtained in all studies, and the current meta-analysis was evaluated three items: serum TG, TB, and glucose. Episodes of hypertriglyceridemia were counted in only two studies: Alburaki et al. [9] defined hypertriglyceridemia as serum TG level > 2.8 mmol/L (247.8 mg/dL), and Drenckpohl et al. [23] as more than 200 mg/dL of serum TG. A meta-analysis of these two studies revealed a significant effect of early high lipid introduction on episodes of hypertriglyceridemia; hypertriglyceridemia was observed more often in the intervention group (RR: 2.72; 95% CI: 1.15, 6.43; *p* = 0.02; *n* = 183; Figure 4h). Episodes of hypoglycemia were documented in only one study by Alburaki et al. [9]; they defined hypoglycemia as blood sugar level <2.6 mmol/L (46.8 mg/dL) and were not significantly different between groups. In contrast, the incidence of hyperglycemia was noted in two studies [9,17]; however, Alburaki et al. reported that no patient required insulin treatment. Therefore, it was impossible to perform a meta-analysis because only one study remained. No significant effect on the serum bilirubin level of early high IVLE administration was found in the meta-analysis of five studies [9,18,19,21,24] (*p* = 0.84 and *I*^2^ = 96%). There was considerably high heterogeneity with a lack of consistency in the timing of blood sampling in each study: highest level [9,19], at the 7th day of life [18,21], and mean value during the first week [24]. To improve the *I*^2^ statics, a subgroup meta-analysis including two studies about the effect on the highest serum total bilirubin [9,19] was conducted and showed no significant effect of early high lipid administration (*p* = 0.20, *I*^2^ = 0, supplementary Figure S1).

## 4. Discussion

In this meta-analysis, the efficacy and safety of early high IVLE administration, namely, equal to or more than 1.5 g/kg/day within the first 24 h of life, was evaluated through nine studies and 520 preterm infants. The results suggested several noticeable findings: early lipid initiation and increasing to high dose immediately in preterm infants seems not only better for growth but also safe. Through the intervention of early high IVLE administration, the mean postnatal weight loss decreased, the mean body weight and mean head circumference near TEA increased, and the incidence of EUGR decreased. Among the secondary outcomes, the incidence of ROP was reduced with early high lipids.

According to previous meta-analyses, the early initiation of IVLE did not result in advantages in terms of growth and did not generate a higher incidence of adverse outcomes [4,5]. In contrast, in the current meta-analysis, based on whether a high dose was achieved within 24 h after birth, there were advantages in terms of growth. The mean maximal percentage of postnatal weight loss was significantly lower in the treatment group, and body weight and head circumference measured near TEA were significantly higher and larger, respectively, in the early high lipid group than in the control group. The PMA at which individual studies measured anthropometrics varied; therefore, the meaning of the result should be interpreted cautiously. However, one of the most consequential findings in our meta-analysis was that the incidence of EUGR calculated considering the corrected age favored early high IVLE introduction for the growth of preterm infants; the incidence of EUGR was 0.27 times lower in the treatment group. There were two perspectives on the major differences from previous meta-analyses. First, the intervention evaluated in this meta-analysis was to assess the preservation of the physiologic intrauterine environment after birth; in other words, whether a high lipid dose (equal to or more than 1.5 g/kg/day) was reached within 24 h after birth. Second, their definitions of early lipids were later than ours: a 5-day difference and 2-day difference in the initiation of lipids: in the study of Simmer et al. [4] and Vlaardingerbroek et al. [5], respectively.

During the last trimester of pregnancy, a brain growth spurt occurs in the human fetus with considerable lipid accretion [27], and appropriate lipid supplementation is crucial to preterm infants exposed to the extrauterine environment in this period. From a cohort study of mothers and infants in Ireland, AA Geraghty and colleagues reported that the serum level of total cholesterol and TG measured in cord blood at delivery were 40.71 mg/dL and 65.74 mg/dL, respectively [28]. The fetus receives a substantial amount of lipids from the mother before delivery; however, these are suddenly discontinued after birth. Because of continuity with the intrauterine environment, high lipid supplementation immediately after birth is a more physiological method. Additionally, a sufficient amount of energy supplementation in the critical period might help long-term neurodevelopment and growth. Previously, Stephens et al. said that increased first-week protein and energy intakes are positively associated with higher scores in developmental tests and negatively associated with growth at 18 months [29], and Roelants et al. reported that more infants scored under datum in the control group than infants in the standard amino acid and soybean oil fat emulsion supplementation group [20].

The early high IVLE treatment did not have significant effect on secondary outcomes: mortality; admission length; and incidence of BPD, NEC, sepsis, IVH, and serum TB level. The results of our meta-analysis were in agreement with several previous meta-analyses [4,5,30,31]. The current meta-analysis of the effects of early high IVLE administration on episodes of hypertriglyceridemia favored the control groups. Correani et al. reported that, with close monitoring of hypertriglyceridemia and IVLE titration at TG levels > 250 mg/dL, infants whose TG level measured >250 mg/dL during early postnatal period did not have growth, disease, and neurodevelopmental disadvantages, unlike the control group [32]. Therefore, high IVLE administration appears to be safe for preterm infants with regular TG monitoring and IVLE titration.

Another interesting finding in our meta-analysis is that early high IVLE showed a favored effect on the incidence of ROP. A recently published RCT reported that enteral arachidonic acid and docosahexaenoic acid supplementation lowered the risk of severe ROP by 50% [33], which is in line with our meta-analysis results. One possible explanation of this result is that infants with early high IVLE might receive an additional source of long-chain polyunsaturated fatty acids in the early postnatal period, and fatty acids could be used for retinal development. Consequently, early high lipid administration may play a key role in reducing the incidence of ROP in preterm infants.

The objective of our meta-analysis, which was to compare “early high” lipids to a control treatment, was different from those of previous studies, and the RCTs included in the current meta-analysis were not duplicated. Simmer et al. [4] and Vlaardingerbroek et al. [5] included a total of seven RCTs [34,35,36,37,38,39,40]. In the study of Gilbertson et al., infants in the “early” lipid group received IVLE from 1 g/kg/day on day 1 to 3 g/kg/day on day 4 [39], and in the studies of Sosenko et al., infants were assigned to the “early” lipid group starting IVLE at less than 12 h, at 0.5 g/kg/day for the first 24 h with an increment of 0.5 g/kg/24 h until 1.5 g/kg was reached [40]. In contrast, because we defined the intervention group as the “early high” lipid group that reached a lipid dose equal to or greater than 1.5 mg/kg/day within the first 24 h, we excluded the RCTs included in previous meta-analyses. The RCT by Tan and Cooke was also excluded for the same reason [41].

The main strength of the current study was that we considered the importance of achieving a high administration dose early as well as early initiation of IVLE, which was distinctly different from previous meta-analyses. We performed a systematic review setting with only patients and interventions in a broad search. In addition, to obtain a high level of evidence, only RCTs were included. However, there were a few limitations of the current meta-analysis. First, there were concerns about the effect of the type of lipid on growth and adverse outcomes. The IVLE used in the RCTs included in our meta-analysis was soybean-based oil emulsion in large part, but Dongming et al. did not describe the exact name of lipid emulsion [17], and Vlaardingerbroek et al. used both soybean-based oil emulsions and SMOF lipid emulsion in the intervention group [19]. The second limitation of the current study was the effects of amino acids. There were differences in protein supplementation dose between early high IVLE groups and control groups in all included studies, which may have acted as a confounding factor to the effect of lipids on growth and adverse outcomes. According to ESPGHAN guidelines on parenteral nutrition [42], in preterm infants, the amino acid supply should start on the first day of life with at least 1.5 g/kg/day to achieve an anabolic state and be increased up to between 2.5 g/kg/day and 3.5 g/kg/day from postnatal day 2. Seven RCTs of the included current meta-analysis satisfied current recommendations; however, in the study of Dongming et al., the infants in the control group were treated with only glucose infusion within 3 days after birth [17], resulting in nutritional deficits. In addition, the types of amino acids given to the preterm infants were different.

## 5. Conclusions

In conclusion, proactive IVLE at high doses is tolerated by preterm infants immediately after birth, and they did not show a significantly increased risk of adverse metabolic outcomes, such as hypertriglyceridemia, hypoglycemia, and hypoglycemia. In addition, there were certain beneficial effects on growth; therefore, early initiation with a high dose of IVLE should be recommended for preterm infants. Large-scale RCTs in preterm infants are prerequisite for determining the optimum dose of IVLE in the first 24 h, the type of IVLE that should be used, and the long-term benefits of early high lipid administration. We would suggest the early initiation with a high dose of IVLE should be recommended for preterm infants.

## Figures and Tables

**Table 1:** Characteristics of studies included in the meta-analysis.

Study	Characteristics					Intervention (IV Lipid Intake)	
	Country	Study Period	Design	Population	* n *	Experimental Group	Control Group
Alburaki, 2020 [9]	Canada	Aug 2018 to Oct 2019	RCT	PT infants with BW < 1500 g and <32 weeks GA	83	Started 2 g/kg/day within 12 h of birth, increased to 3 g/kg/day the next day	Started 0.5 g/kg/day (BW < 1000 g) or 1 g/kg/day (BW ≥ 1000 g) between 12–24 h, and advancing to 3 g/kg/day (increment 0.5 g/kg/day)
Dongming 2016 [17]	China	June 2013 to June 2015	RCT	PT infants with BW < 1500 g	80	Started 1.5 g/kg/day within 24 h of birth and advancing to 3 g/kg/day (increment, 0.5 g/kg/day)	Only glucose within 3 days and started same parenteral nutrition after day 3.
Tagare, 2013 [18]	India	Oct 2009 to Mar 2010	RCT	PT infants with BW < 1500 g and <32 weeks GA	34	Started with 2 g/kg/day within 24 h of birth, remained same thereafter	Started 1 g/kg/day at day 3, remained same thereafter
Vlaardingerbroek, 2013 [19]	Netherland	Dec 2008 to Jan 2012	RCT	PT infants with BW < 1500 g	97 ^1^	Started 2 g/kg/day immediately advancing to 3 g/kg/day, day 2.	Started 1.4 g/kg/day at day 2, next day increased to 2.8 g/kg/day
Bulbul, 2012 [21]	Turkey		RCT	PT infants with 750 g < BW < 1500 g and <32 weeks GA	41	Started 3 g/kg/day, on day 1, remained same thereafter	Started 1.0 g/kg/day at day 3, advancing up to 3 g/kg/day (increment 1.0 g/kg/day)
Can, 2012 [22]	Turkey	Feb 2009 to May 2010	RCT	PT infants with <34 weeks GA	53	Started 2 g/kg/day on day 1, advancing to 3.0 g/kg/day on day 2.	Started 1.0 g/kg/day on day 1, advancing up to 3 g/kg/day (increment 1.0 g/kg/day)
Drenckpohl, 2008 [23]	Illinois, US	June 2005 to Sep 2009	RCT	PT infants with 750 g < BW < 1500 g	100	Started 2 g/kg/day on day 1, advancing to 3 g/kg/day (increment 0.5 g/kg/day)	Started 0.5 g/kg/day on day 1, advancing up to 3 g/kg/day (increment 0.5 g/kg/day)
Ibranhim, 2004 [24]	Louisiana, US	July 2001 to Apr 2002	RCT	PT infants with 500 g < BW < 1250 g and 24 ≤ GA < 32 weeks	32	Started 3 g/kg/day within 2 h after birth, remained same thereafter	started 0.5 g/kg/day at 48 h after birth, advancing up to 3 g/kg/day (increment 0.5 g/kg/day)

RCT, randomized controlled trial; PT, preterm; BW, birth weight; GA, gestational age; VLBW very low birth weight; AA, aminoacid; ^1^ Vlaardingerbroek et al. included 144 VLBW infants: 48 in the control group, 49 in the AA + lipid group, and 47 in the high AA + lipid group. In the current meta-analysis, we analyzed the AA + lipid group and the control group.

**Table 2:** Baseline characteristics and outcome measures of infants in studies analyzed.

First Author, Year	*n*	Male	GA	BW	Wt Gain Rate	Max %Age of wt Loss	Time to Regain BW	Wt Near TEA	HC Near TEA	EUGR	Death	NICU Stay	BPD/CLD	NEC ≥ 2	Proven Sepsis	IVH ≥ 3	ROP	HyperTG	Hypoglycemia	Hyperglycemia	Serum TB
		n (%)	wk	g	g/kg/day	%	Day	g	cm	n (%)	n	day	n (%)	n (%)	n (%)	n (%)	n (%)	n (%)	n (%)	n (%)	mg/dL
***Intervention***																					
Alburaki, 2020 [9]	45	28 (62)	27.1 ± 2.3	1019 ± 271	15.2 ± 2.0 ^1^	10.4 ± 3.6 ^2^	10.5 (8,13)	2278 ± 303 ^3^	31.3 ± 1.5 ^3^	17 (38.6) ^3^	1	82 (49.5,107)	15 (33.3) ^4^	1 (2.2)	3 (6.7)	4 (8.9)	6 (25) ^5^	15 (33.3) ^6^	14 (31.1) ^7^	0 ^8^	8.42 ± 1.52 ^9^
Dongming, 2016 [17]	40	24 (60)	30.2 (28,34)	1140 ± 220	-	7.7 ± 1.5	8.2 ± 2.4	-	-	-	-	-	-	-	-	-	-	-	-	1	-
Tagare, 2013 [18]	17		30.5 ± 2.6	1162 ± 224	-	-	9.5 ± 6.7	-	-	-	2	19.5 ± 13.3	-	-	-	-	-	-	-	-	6.7 ± 3.1 ^10^
Vlaardingerbroek, 2013 [19]	49	19 (39)	27.2 ± 2.2	876 ± 209	25.0 ± 5.2 ^11^	-	8 (5,12), ns	-	-	-	10	86.5 ± 29.1	11 (22) ^12^	4 (8)	17 (35)	2 (4)	0 (0)	27% ^13^	-	24% ^8^	8.26 ± 2.2 ^9^
Bulbul, 2012 [21]	22	70%	29.1 ± 1.1	1316 ± 247	-	-	12.5 ± 5.4	2210 ± 91 ^14^	32.1 ± 2.3 ^14^	-	-	34.4 ± 18.1	-	2 (9)	1 (4.5)	0	-	-	-	-	7.2 ± 0.6 ^10^
Can, 2012 [22]	25	16 (64)	31.3 (27,33)	1622 ± 276	-	-	12.7 ± 2.8	3180 ± 474 ^15^	34.7 ± 1.5 ^15^	13 (52) ^15^	1	28 ± 15.6	1 (4) ^16^	1 (4)	-	-	2 (8) ^17^	-	-	-	-
Drenckpohl, 2008 [23]	48	58.3%	28.8 ± 1.7	1182 ± 198	-	-	12.5 ± 3.7	1894 ± 392 ^14^	30.9 ± 2.2 ^14^	28 (58) ^14^	0	43.65 ± 19	4 (8) ^16^	0 (0)	-	11 (23) ^18^	3 (6) ^19^	7 (15) ^20^	-	0 % ^8^	-
Ibranhim, 2004 [24]	16	10 (63)	27 ± 1.6	846 ± 261	-	-	-	-	-	-	1	-	9 (56.25) ^16^	-	6	4	2	-	-	-	7.7 ± 0.42 ^21^
***Control***																					
Alburaki, 2020 [9]	38	25 (66)	27.3 ± 2.4	1011 ± 250	15.3 ± 3.5 ^1^	12.7 ± 4.6 ^2^	11.5 (8,16)	2165 ± 301 ^3^	30.5 ± 1.4 ^3^	25 (67.6) ^3^	1	80.5 (58,96)	8 (21.1) ^4^	1 (2.6)	2 (5.3)	2 (5.3)	6 (26.1) ^5^	7 (18.4) ^6^	11 (28.9) ^7^	0^8^	8.65 ± 1.7 ^9^
Dongming, 2016 [17]	40	25 (63)	30.4 (28,34)	1148 ± 216	-	10.6 ± 3.3	11.6 ± 3.0	-	-	-	-	-	-	-	-	-	-	-	-	3	-
Tagare, 2013 [18]	17		32.1 ± 2.8	1264 ± 194	-	-	11.5 ± 6.7	-	-	-	1	20.2 ± 12.9	-	-	-	-	-	-	-	-	7.2 ± 2.5 ^10^
Vlaardingerbroek, 2013 [19]	48	25 (52)	27.8 ± 2.3	843 ± 224	25.8 ± 8.1 ^11^	-	8 (5,12), ns	-	-	-	5	91.0 ± 39.9	16 (33) ^12^	2 (4)	8 (17)	1 (2)	2 (4)	44% ^13^	-	6% ^8^	8.77 ± 1.88 ^9^
Bulbul, 2012 [21]	22	52%	29.4 ± 1.8	1355 ± 237	-	-	10.2 ± 3.9	2155 ± 180 ^14^	31.2 ± 2.1 ^14^	-	-	33.5 ± 19.4	-	1 (4.5)	1 (4.5)	0	-	-	-	-	7.7 ± 0.5 ^10^
Can, 2012 [22]	25	15 (60)	31.4 (27,33)	1598 ± 346	-	-	14.2 ± 3.0	2992 ± 445 ^15^	33.6 ± 1.5 ^15^	22 (88) ^15^	2	29.9 ± 4.8	1 (4)	2 (8)	-	-	8 (32) ^17^	-	-	-	-
Drenckpohl, 2008 [23]	52	55.8%	28.6 ± 1.8	1134 ± 223	-	-	12.9 ± 3.8	1946 ± 771 ^14^	31 ± 2 ^14^	43 (83) ^14^	3	50.58 ± 33	7 (14) ^16^	7 (14)	-	11 (21) ^18^	12 (23) ^19^	2 (4) ^20^	-	10 % ^8^	-
Ibranhim, 2004 [24]	16	9 (56)	26.8 ± 1.5	968 ± 244	-	-	-	-	-	-	2	-	8 (50) ^16^	-	7	5	3	-	-	-	6.2 ± 0.43 ^21^

Values are means ± standard deviation, median (IQR) or frequencies (percentage), as appropriate. GA, gestational age; BW, birth weight; Wt, weight; TEA, term equivalent age; EUGR, extrauterine growth restriction; NICU, neonatal intensive care unit; BPD, bronchopulmonary dysplasia; CLD, chronic lung disease; NEC, necrotizing enterocolitis; IVH, intraventricular hemorrhage; ROP, retinopathy of prematurity; HyperTG, hypertriglyceridemia; TB, total bilirubin; -, no data; ns, no data, only described as “not significant”; ^1^ weight gain from regaining birth weight to 36 PMA(postmenstrual age); ^2^ calculated using the difference between birth weight and lowest postnatal weight in the first 2 weeks of life; ^3^ at 36 PMA; ^4^ incidence of, according to Child Health and Human Development, the requirement for positive pressure support or oxygen dependency at 36 PMA; ^5^ retinopathy of prematurity as stage 2 or higher or requiring treatment; ^6^ hypertriglyceridemia >2.8 mmol/L, sampling was performed after 24 h of lipid increment to 3 g/kg/day; ^7^ hypoglycemia, serum glucose <2.6 mmol/L; ^8^ hyperglycemia, requiring insulin treatment; ^9^ highest total bilirubin level; ^10^ obtained on day 7; ^11^ until discharge home or term equivalent age, whichever came first; ^12^ Diagnosed at 36 PMA as determined by the physiologic definition with an oxygen reduction test; ^13^ Hypertriglyceridemia, TG level > 3 mmol/L (265 mg/dL); ^14^ at discharge; ^15^ at term equivalent age; ^16^ Need for oxygen at 36 PMA; ^17^ defined as threshold ROP or higher stages of ROP according to indirect ophthalmoscopy; ^18^ IVH all grade; ^19^ ROP, Vermont oxford criteria; ^20^ hyperTG ≥ 201 mg/dL; ^21^ Mean, during the first 7 days.

## Data Availability

Data is contained within the article or supplementary material.

## Supplementary Materials

The following are available online at https://www.mdpi.com/article/10.3390/nu13051535/s1, Figure S1: Subgroup meta-analysis of the effects on clinical outcome and morbidities of early high IVLE. IVLE, intravenous lipid emulsion; IV, inverse variance; M-H, CI, confidence interval.
