# Supplementary material for: Safety and Efficacy of Early High Parenteral Lipid Supplementation in Preterm Infants: A Systematic Review and Meta-Analysis
Source: Nutrients. 2021 May 2;13(5):1535. doi: 10.3390/nu13051535 (PMC8147506; doi:10.3390/nu13051535)
Supplement: Supplementary file 1 [file nutrients-13-01535-s001.zip › Supplemental file (S1).pdf]

Search strategies for database searching

PubMed, EMBASE, and Cochrane were searched using separate comprehensive search strategies.

Search strategies

PubMed

Number	Search query	Results
#1	"Infant, Premature"[Mesh]	56,391
#2	"Infant, Premature"[TW] OR "Infants, Premature"[TW] OR "Premature Infant"[TW] OR "Preterm Infants"[TW] OR "Infant, Preterm"[TW] OR "Infants, Preterm"[TW] OR "Preterm Infant"[TW] OR "Premature Infants"[TW] OR "Neonatal Prematurity"[TW] OR "Prematurity, Neonatal"[TW] OR "Premature"[TW]	198,979
#3	"Infant, Extremely Premature"[Mesh]	2,604
#4	"Infant, Extremely Premature"[TW] OR "Extremely Premature Infant"[TW] OR "Infants, Extremely Premature"[TW] OR "Premature Infant, Extremely"[TW] OR "Premature Infants, Extremely"[TW] OR "Extremely Preterm Infants"[TW] OR "Extremely Preterm Infant"[TW] OR "Infant, Extremely Preterm"[TW] OR "Infants, Extremely Preterm"[TW] OR "Preterm Infant, Extremely"[TW] OR "Preterm Infants, Extremely"[TW] OR "Extremely Premature Infants"[TW]	3,947
#5	"Premature Birth"[Mesh]	14,129
#6	"Premature Birth"[TW] OR "Birth, Premature"[TW] OR "Births, Premature"[TW] OR "Premature Births"[TW] OR "Preterm Birth"[TW] OR "Birth, Preterm"[TW] OR "Births, Preterm"[TW] OR "Preterm Births"[TW] OR "Preterm"[TW] OR "Very Preterm Infants"[TW] OR "Very Preterm Infant"[TW]	82,384
#7	"Infant, Low Birth Weight"[Mesh]	34,621
#8	"Infant, Low Birth Weight"[TW] OR "Low-Birth-Weight Infant"[TW] OR "Infant, Low-Birth-Weight"[TW] OR "Infants, Low-Birth-Weight"[TW] OR "Low Birth Weight Infant"[TW] OR "Low-Birth-Weight Infants"[TW] OR "Low Birth Weight"[TW] OR "Birth Weight, Low"[TW] OR "Birth Weights, Low"[TW] OR "Low Birth Weights"[TW]	42,085
#9	"Infant, Very Low Birth Weight"[Mesh]	10,255
#10	"Infant, Very Low Birth Weight"[TW] OR "Very-Low-Birth-Weight Infant"[TW] OR "Infant, Very-Low-Birth-Weight"[TW] OR "Infants, Very-Low-Birth-Weight"[TW] OR "Very Low Birth Weight Infant"[TW] OR "Very-Low-Birth-Weight Infants"[TW] OR "Very Low Birth Weight"[TW]	12,114
#11	"Infant, Extremely Low Birth Weight"[Mesh]	1,906
#12	"Infant, Extremely Low Birth Weight"[TW] OR "Extremely Low Birth Weight Infant"[TW]	2,053

#13 Combine	(((((((((("Infant, Premature"[Mesh]) OR ("Infant, Premature"[TW] OR "Infants, Premature"[TW] OR "Premature Infant"[TW] OR "Preterm Infants"[TW] OR "Infant, Preterm"[TW] OR "Infants, Preterm"[TW] OR "Preterm Infant"[TW] OR "Premature Infants"[TW] OR "Neonatal Prematurity"[TW] OR "Prematurity, Neonatal"[TW] OR "Premature"[TW])) OR ("Infant, Extremely Premature"[Mesh])) OR ("Infant, Extremely Premature"[TW] OR "Extremely Premature Infant"[TW] OR "Infants, Extremely Premature"[TW] OR "Premature Infant, Extremely"[TW] OR "Premature Infants, Extremely"[TW] OR "Extremely Preterm Infants"[TW] OR "Extremely Preterm Infant"[TW] OR "Infant, Extremely Preterm"[TW] OR "Infants, Extremely Preterm"[TW] OR "Preterm Infant, Extremely"[TW] OR "Preterm Infants, Extremely"[TW] OR "Extremely Premature Infants"[TW])) OR ("Premature Birth"[Mesh])) OR ("Premature Birth"[TW] OR "Birth, Premature"[TW] OR "Births, Premature"[TW] OR "Premature Births"[TW] OR "Preterm Birth"[TW] OR "Birth, Preterm"[TW] OR "Births, Preterm"[TW] OR "Preterm Births"[TW] OR "Preterm"[TW] OR "Very Preterm Infants"[TW] OR "Very Preterm Infant"[TW])) OR ("Infant, Low Birth Weight"[Mesh])) OR ("Infant, Low Birth Weight"[TW] OR "Low-Birth-Weight Infant"[TW] OR "Infant, Low-Birth-Weight"[TW] OR "Infants, Low-Birth-Weight"[TW] OR "Low Birth Weight Infant"[TW] OR "Low-Birth-Weight Infants"[TW] OR "Low Birth Weight"[TW] OR "Birth Weight, Low"[TW] OR "Birth Weights, Low"[TW] OR "Low Birth Weights"[TW])) OR ("Infant, Very Low Birth Weight"[Mesh])) OR ("Infant, Very Low Birth Weight"[TW] OR "Very-Low-Birth-Weight Infant"[TW] OR "Infant, Very-Low-Birth-Weight"[TW] OR "Infants, Very-Low-Birth-Weight"[TW] OR "Very Low Birth Weight Infant"[TW] OR "Very-Low-Birth-Weight Infants"[TW] OR "Very Low Birth Weight"[TW])) OR ("Infant, Extremely Low Birth Weight"[Mesh])) OR ("Infant, Extremely Low Birth Weight"[TW] OR "Extremely Low Birth Weight Infant"[TW])	243,169
#14	"Lipids"[Mesh]	1,165,798
#15	"Lipids"[TW] OR "high lipid infusion"[TW] OR "high lipid"[TW] OR "lipid infusion"[TW] OR "lipid emulsion"[TW] OR "High Early Parenteral Lipid"[TW]	238,976
#16	"Fatty Acids"[Mesh]	456,416
#17	"Fatty Acids"[TW] OR "Acids, Fatty"[TW] OR "Fatty Acids, Esterified"[TW] OR "Acids, Esterified Fatty"[TW] OR "Esterified Fatty Acids"[TW] OR "Fatty Acids, Saturated"[TW] OR "Acids, Saturated Fatty"[TW] OR "Saturated Fatty Acids"[TW] OR "Aliphatic Acids"[TW] OR "Acids, Aliphatic"[TW]	215,477
#18	"Infusions, Intravenous"[Mesh]	55,101
#19	"Infusions, Intravenous"[TW] OR "Intravenous Infusions"[TW] OR "Infusion, Intravenous"[TW] OR "Intravenous Infusion"[TW] OR "Intravenous Drip"[TW] OR "Drip, Intravenous"[TW] OR "Drip Infusions"[TW] OR "Drip Infusion"[TW] OR "Infusion, Drip"[TW] OR "Infusions, Drip"[TW] OR "intravenous"[TW] OR "intravenous lipid emulsion"[TW]	396,527
#20	"Infusions, Parenteral"[Mesh]	92,554
#21	"Infusions, Parenteral"[TW] OR "Parenteral Infusions"[TW] OR "Infusion, Parenteral"[TW] OR "Parenteral Infusion"[TW] OR "Intra-Abdominal Infusions"[TW] OR "Infusion, Intra-Abdominal"[TW] OR "Infusions, Intra-Abdominal"[TW] OR "Intra Abdominal Infusions"[TW] OR "Intra-Abdominal Infusion"[TW] OR "Peritoneal Infusions"[TW] OR "Infusion, Peritoneal"[TW] OR "Infusions, Peritoneal"[TW] OR "Peritoneal Infusion"[TW] OR "Intraperitoneal Infusions"[TW] OR "Infusion, Intraperitoneal"[TW] OR "Infusions, Intraperitoneal"[TW] OR "Intraperitoneal Infusion"[TW] OR "parenteral"[TW]	84,442
#22	"Parenteral Nutrition"[Mesh]	24,064
#23	"Parenteral Nutrition"[TW] OR "Nutrition, Parenteral"[TW] OR "Parenteral Feeding"[TW] OR "Feeding, Parenteral"[TW] OR "Feedings, Parenteral"[TW] OR "Parenteral Feedings"[TW] OR "Intravenous Feeding"[TW] OR "Feeding, Intravenous"[TW] OR "Feedings, Intravenous"[TW] OR "Intravenous Feedings"[TW]	32,451

#24 Combine	((((((((("Lipids"[Mesh]) OR ("Lipids"[TW] OR "high lipid infusion"[TW] OR "high lipid"[TW] OR "lipid infusion"[TW] OR "lipid emulsion"[TW] OR "High Early Parenteral Lipid"[TW])) OR ("Fatty Acids"[Mesh])) OR ("Fatty Acids"[TW] OR "Acids, Fatty"[TW] OR "Fatty Acids, Esterified"[TW] OR "Acids, Esterified Fatty"[TW] OR "Esterified Fatty Acids"[TW] OR "Fatty Acids, Saturated"[TW] OR "Acids, Saturated Fatty"[TW] OR "Saturated Fatty Acids"[TW] OR "Aliphatic Acids"[TW] OR "Acids, Aliphatic"[TW])) OR ("Infusions, Intravenous"[Mesh])) OR ("Infusions, Intravenous"[TW] OR "Intravenous Infusions"[TW] OR "Infusion, Intravenous"[TW] OR "Intravenous Infusion"[TW] OR "Intravenous Drip"[TW] OR "Drip, Intravenous"[TW] OR "Drip Infusions"[TW] OR "Drip Infusion"[TW] OR "Infusion, Drip"[TW] OR "Infusions, Drip"[TW] OR "intravenous"[TW] OR "intravenous lipid emulsion"[TW])) OR ("Infusions, Parenteral"[Mesh])) OR ("Infusions, Parenteral"[TW] OR "Parenteral Infusions"[TW] OR "Infusion, Parenteral"[TW] OR "Parenteral Infusion"[TW] OR "Intra-Abdominal Infusions"[TW] OR "Infusion, Intra-Abdominal"[TW] OR "Infusions, Intra-Abdominal"[TW] OR "Intra Abdominal Infusions"[TW] OR "Intra-Abdominal Infusion"[TW] OR "Peritoneal Infusions"[TW] OR "Infusion, Peritoneal"[TW] OR "Infusions, Peritoneal"[TW] OR "Peritoneal Infusion"[TW] OR "Intraperitoneal Infusions"[TW] OR "Infusion, Intraperitoneal"[TW] OR "Infusions, Intraperitoneal"[TW] OR "Intraperitoneal Infusion"[TW] OR "parenteral"[TW])) OR ("Parenteral Nutrition"[Mesh])) OR ("Parenteral Nutrition"[TW] OR "Nutrition, Parenteral"[TW] OR "Parenteral Feeding"[TW] OR "Feeding, Parenteral"[TW] OR "Feedings, Parenteral"[TW] OR "Parenteral Feedings"[TW] OR "Intravenous Feeding"[TW] OR "Feeding, Intravenous"[TW] OR "Feedings, Intravenous"[TW] OR "Intravenous Feedings"[TW])	1,697,412
#25 Combine	#13 AND #24	19,052
#26 Limit	#25 AND ("Randomized Controlled Trial" [Publication Type] OR "Controlled Clinical Trial" [Publication Type] OR "Randomized Controlled Trials as Topic"[Mesh] OR "Random Allocation"[Mesh] OR "Double-Blind Method"[Mesh] OR "Single-Blind Method"[Mesh] OR "Clinical Trial" [Publication Type] OR "Clinical Trials as Topic"[Mesh] OR "Clinical Trial"[TW] OR ((singl*[TW] OR doubl*[TW] OR trebl*[TW] OR tripl*[TW]) AND (mask*[TW] OR blind*[TW])) OR "Placebos"[Mesh] OR placebo*[TW] OR random*[TW] OR "Research Design"[Mesh:NoExp])	3,603

EMBASE

Number	Search query	Results
#1	"prematurity"/exp	112,379
#2	"Infant, Premature":ti,ab,kw,de OR "Infants, Premature":ti,ab,kw,de OR "Premature Infant":ti,ab,kw,de OR "Preterm Infants":ti,ab,kw,de OR "Infant, Preterm":ti,ab,kw,de OR "Infants, Preterm":ti,ab,kw,de OR "Preterm Infant":ti,ab,kw,de OR "Premature Infants":ti,ab,kw,de OR "Neonatal Prematurity":ti,ab,kw,de OR "Prematurity, Neonatal":ti,ab,kw,de OR "Premature":ti,ab,kw,de	238,482
#3	"Infant, Extremely Premature":ti,ab,kw,de OR "Extremely Premature Infant":ti,ab,kw,de OR "Infants, Extremely Premature":ti,ab,kw,de OR "Premature Infant, Extremely":ti,ab,kw,de OR "Premature Infants, Extremely":ti,ab,kw,de OR "Extremely Preterm Infants":ti,ab,kw,de OR "Extremely Preterm Infant":ti,ab,kw,de OR "Infant, Extremely Preterm":ti,ab,kw,de OR "Infants, Extremely Preterm":ti,ab,kw,de OR "Preterm Infant, Extremely":ti,ab,kw,de OR "Preterm Infants, Extremely":ti,ab,kw,de OR "Extremely Premature Infants":ti,ab,kw,de	2,766

#4	"Premature Birth":ti,ab,kw,de OR "Birth, Premature":ti,ab,kw,de OR "Births, Premature":ti,ab,kw,de OR "Premature Births":ti,ab,kw,de OR "Preterm Birth":ti,ab,kw,de OR "Birth, Preterm":ti,ab,kw,de OR "Births, Preterm":ti,ab,kw,de OR "Preterm Births":ti,ab,kw,de OR "Preterm":ti,ab,kw,de OR "Very Preterm Infants":ti,ab,kw,de OR "Very Preterm Infant":ti,ab,kw,de	112,378
#5	"low birth weight"/exp	62,516
#6	"Infant, Low Birth Weight":ti,ab,kw,de OR "Low-Birth-Weight Infant":ti,ab,kw,de OR "Infant, Low-Birth-Weight":ti,ab,kw,de OR "Infants, Low-Birth-Weight":ti,ab,kw,de OR "Low Birth Weight Infant":ti,ab,kw,de OR "Low-Birth-Weight Infants":ti,ab,kw,de OR "Low Birth Weight":ti,ab,kw,de OR "Birth Weight, Low":ti,ab,kw,de OR "Birth Weights, Low":ti,ab,kw,de OR "Low Birth Weights":ti,ab,kw,de	58,660
#7	"very low birth weight"/exp	15,117
#8	"Infant, Very Low Birth Weight":ti,ab,kw,de OR "Very-Low-Birth-Weight Infant":ti,ab,kw,de OR "Infant, Very-Low-Birth-Weight":ti,ab,kw,de OR "Infants, Very-Low-Birth-Weight":ti,ab,kw,de OR "Very Low Birth Weight Infant":ti,ab,kw,de OR "Very-Low-Birth-Weight Infants":ti,ab,kw,de OR "Very Low Birth Weight":ti,ab,kw,de	14,956
#9	"extremely low birth weight"/exp	3,376
#10	"Infant, Extremely Low Birth Weight":ti,ab,kw,de OR "Extremely Low Birth Weight Infant":ti,ab,kw,de	358
#11 Combine	#1 OR #2 OR #3 OR #4 OR #5 OR #6 OR #7 OR #8 OR #9 OR #10	339,138
#12	"lipid"/exp	1,631,386
#13	"Lipids":ti,ab,kw,de OR "high lipid infusion":ti,ab,kw,de OR "high lipid":ti,ab,kw,de OR "lipid infusion":ti,ab,kw,de OR "lipid emulsion":ti,ab,kw,de OR "High Early Parenteral Lipid":ti,ab,kw,de	198,369
#14	"fatty acid"/exp	600,478
#15	"Fatty Acids":ti,ab,kw,de OR "Acids, Fatty":ti,ab,kw,de OR "Fatty Acids, Esterified":ti,ab,kw,de OR "Acids, Esterified Fatty":ti,ab,kw,de OR "Esterified Fatty Acids":ti,ab,kw,de OR "Fatty Acids, Saturated":ti,ab,kw,de OR "Acids, Saturated Fatty":ti,ab,kw,de OR "Saturated Fatty Acids":ti,ab,kw,de OR "Aliphatic Acids":ti,ab,kw,de OR "Acids, Aliphatic":ti,ab,kw,de	171,049
#16	"intravenous drug administration"/exp	383,121

#17	"Infusions, Intravenous":ti,ab,kw,de OR "Intravenous Infusions":ti,ab,kw,de OR "Infusion, Intravenous":ti,ab,kw,de OR "Intravenous Infusion":ti,ab,kw,de OR "Intravenous Drip":ti,ab,kw,de OR "Drip, Intravenous":ti,ab,kw,de OR "Drip Infusions":ti,ab,kw,de OR "Drip Infusion":ti,ab,kw,de OR "Infusion, Drip":ti,ab,kw,de OR "Infusions, Drip":ti,ab,kw,de OR "intravenous":ti,ab,kw,de OR "intravenous lipid emulsion":ti,ab,kw,de OR "administration, intravenous":ti,ab,kw,de OR "drug administration, intravenous":ti,ab,kw,de OR "infusion, intravenous":ti,ab,kw,de OR "infusions, intravenous":ti,ab,kw,de OR "injection, intravenous":ti,ab,kw,de OR "injections, intravenous":ti,ab,kw,de OR "intravenous administration":ti,ab,kw,de OR "intravenous bolus administration":ti,ab,kw,de OR "intravenous bolus drug administration":ti,ab,kw,de OR "intravenous bolus injection":ti,ab,kw,de OR "intravenous dose":ti,ab,kw,de OR "intravenous drip":ti,ab,kw,de OR "intravenous drip administration":ti,ab,kw,de OR "intravenous drip infusion":ti,ab,kw,de OR "intravenous drip injection":ti,ab,kw,de OR "intravenous fluid administration":ti,ab,kw,de OR "intravenous fluid therapy":ti,ab,kw,de OR "intravenous infusion":ti,ab,kw,de OR "intravenous infusions":ti,ab,kw,de OR "intravenous injection":ti,ab,kw,de OR "intravenous injections":ti,ab,kw,de OR "intravenous medication":ti,ab,kw,de OR "intravenous therapy":ti,ab,kw,de OR "intravenous transfusion":ti,ab,kw,de OR "iv administration":ti,ab,kw,de OR "IV drug administration":ti,ab,kw,de OR "IV drug delivery":ti,ab,kw,de OR "IV drug injection":ti,ab,kw,de OR "IV drug therapy":ti,ab,kw,de OR "iv fluid administration":ti,ab,kw,de OR "iv infusion":ti,ab,kw,de OR "iv injection":ti,ab,kw,de OR "IV medication":ti,ab,kw,de OR "iv transfusion":ti,ab,kw,de OR "vein infusion":ti,ab,kw,de OR "vein injection":ti,ab,kw,de OR "venous drip":ti,ab,kw,de OR "venous infusion":ti,ab,kw,de OR "venous injection":ti,ab,kw,de OR "venous transfusion":ti,ab,kw,de	714,962
#18	"parenteral drug administration"/exp	777,606
#19	"Infusions, Parenteral":ti,ab,kw,de OR "Parenteral Infusions":ti,ab,kw,de OR "Infusion, Parenteral":ti,ab,kw,de OR "Parenteral Infusion":ti,ab,kw,de OR "Intra-Abdominal Infusions":ti,ab,kw,de OR "Infusion, Intra-Abdominal":ti,ab,kw,de OR "Infusions, Intra-Abdominal":ti,ab,kw,de OR "Intra Abdominal Infusions":ti,ab,kw,de OR "Intra-Abdominal Infusion":ti,ab,kw,de OR "Peritoneal Infusions":ti,ab,kw,de OR "Infusion, Peritoneal":ti,ab,kw,de OR "Infusions, Peritoneal":ti,ab,kw,de OR "Peritoneal Infusion":ti,ab,kw,de OR "Intraperitoneal Infusions":ti,ab,kw,de OR "Infusion, Intraperitoneal":ti,ab,kw,de OR "Infusions, Intraperitoneal":ti,ab,kw,de OR "Intraperitoneal Infusion":ti,ab,kw,de OR "parenteral":ti,ab,kw,de OR "drug administration, parenteral":ti,ab,kw,de OR "hypodermic drug administration":ti,ab,kw,de OR "infusion, parenteral":ti,ab,kw,de OR "infusions, parenteral":ti,ab,kw,de OR "parenteral administration":ti,ab,kw,de OR "parenteral delivery":ti,ab,kw,de OR "parenteral dosage":ti,ab,kw,de OR "parenteral dose":ti,ab,kw,de OR "parenteral drug delivery":ti,ab,kw,de OR "parenteral drug infusion":ti,ab,kw,de OR "parenteral drug therapy":ti,ab,kw,de OR "parenteral infusion":ti,ab,kw,de OR "parenteral infusions":ti,ab,kw,de OR "parenteral injection":ti,ab,kw,de OR "parenteral medication":ti,ab,kw,de OR "parenteral therapy":ti,ab,kw,de OR "parenteral treatment":ti,ab,kw,de	92,152
#20	"parenteral nutrition"/exp	50,428
#21	"Parenteral Nutrition":ti,ab,kw,de OR "Nutrition, Parenteral":ti,ab,kw,de OR "Parenteral Feeding":ti,ab,kw,de OR "Feeding, Parenteral":ti,ab,kw,de OR "Feedings, Parenteral":ti,ab,kw,de OR "Parenteral Feedings":ti,ab,kw,de OR "Intravenous Feeding":ti,ab,kw,de OR "Feeding, Intravenous":ti,ab,kw,de OR "Feedings, Intravenous":ti,ab,kw,de OR "Intravenous Feedings":ti,ab,kw,de	52,374
#22 Combine	#12 OR #13 OR #14 OR #15 OR #16 OR #17 OR #18 OR #19 OR #20 OR #21	2,803,013
#23 Combine	#11 AND #22	35,621

#24 Limit	#23 AND [randomized controlled trial]/lim	1,876

Cochrane

Number	Search query	Results
#1	[mh "Infant, Premature"]	3,745
#2	"Infant, Premature":ti,ab,kw OR "Infants, Premature":ti,ab,kw OR "Premature Infant":ti,ab,kw OR "Preterm Infants":ti,ab,kw OR "Infant, Preterm":ti,ab,kw OR "Infants, Preterm":ti,ab,kw OR "Preterm Infant":ti,ab,kw OR "Premature Infants":ti,ab,kw OR "Neonatal Prematurity":ti,ab,kw OR "Prematurity, Neonatal":ti,ab,kw OR "Premature":ti,ab,kw	20,485
#3	[mh "Infant, Extremely Premature"]	186
#4	"Infant, Extremely Premature":ti,ab,kw OR "Extremely Premature Infant":ti,ab,kw OR "Infants, Extremely Premature":ti,ab,kw OR "Premature Infant, Extremely":ti,ab,kw OR "Premature Infants, Extremely":ti,ab,kw OR "Extremely Preterm Infants":ti,ab,kw OR "Extremely Preterm Infant":ti,ab,kw OR "Infant, Extremely Preterm":ti,ab,kw OR "Infants, Extremely Preterm":ti,ab,kw OR "Preterm Infant, Extremely":ti,ab,kw OR "Preterm Infants, Extremely":ti,ab,kw OR "Extremely Premature Infants":ti,ab,kw	507
#5	[mh "Premature Birth"]	1,453
#6	"Premature Birth":ti,ab,kw OR "Birth, Premature":ti,ab,kw OR "Births, Premature":ti,ab,kw OR "Premature Births":ti,ab,kw OR "Preterm Birth":ti,ab,kw OR "Birth, Preterm":ti,ab,kw OR "Births, Preterm":ti,ab,kw OR "Preterm Births":ti,ab,kw OR "Preterm":ti,ab,kw OR "Very Preterm Infants":ti,ab,kw OR "Very Preterm Infant":ti,ab,kw	13,895
#7	[mh "Infant, Low Birth Weight"]	2,175
#8	"Infant, Low Birth Weight":ti,ab,kw OR "Low-Birth-Weight Infant":ti,ab,kw OR "Infant, Low-Birth-Weight":ti,ab,kw OR "Infants, Low-Birth-Weight":ti,ab,kw OR "Low Birth Weight Infant":ti,ab,kw OR "Low-Birth-Weight Infants":ti,ab,kw OR "Low Birth Weight":ti,ab,kw OR "Birth Weight, Low":ti,ab,kw OR "Birth Weights, Low":ti,ab,kw OR "Low Birth Weights":ti,ab,kw	4,952
#9	[mh "Infant, Very Low Birth Weight"]	954
#10	"Infant, Very Low Birth Weight":ti,ab,kw OR "Very-Low-Birth-Weight Infant":ti,ab,kw OR "Infant, Very-Low-Birth-Weight":ti,ab,kw OR "Infants, Very-Low-Birth-Weight":ti,ab,kw OR "Very Low Birth Weight Infant":ti,ab,kw OR "Very-Low-Birth-Weight Infants":ti,ab,kw OR "Very Low Birth Weight":ti,ab,kw	2,201
#11	[mh "Infant, Extremely Low Birth Weight"]	118
#12	"Infant, Extremely Low Birth Weight":ti,ab,kw OR "Extremely Low Birth Weight Infant":ti,ab,kw	123
#13 Combine	{OR #1-#12}	25,845
#14	[mh "Lipids"]	48,350
#15	"Lipids":ti,ab,kw OR "high lipid infusion":ti,ab,kw OR "high lipid":ti,ab,kw OR "lipid infusion":ti,ab,kw OR "lipid emulsion":ti,ab,kw OR "High Early Parenteral Lipid":ti,ab,kw	17,168
#16	[mh "Fatty Acids"]	22,257

#17	"Fatty Acids":ti,ab,kw OR "Acids, Fatty":ti,ab,kw OR "Fatty Acids, Esterified":ti,ab,kw OR "Acids, Esterified Fatty":ti,ab,kw OR "Esterified Fatty Acids":ti,ab,kw OR "Fatty Acids, Saturated":ti,ab,kw OR "Acids, Saturated Fatty":ti,ab,kw OR "Saturated Fatty Acids":ti,ab,kw OR "Aliphatic Acids":ti,ab,kw OR "Acids, Aliphatic":ti,ab,kw	13,306
#18	[mh "Infusions, Intravenous"]	10,248
#19	"Infusions, Intravenous":ti,ab,kw OR "Intravenous Infusions":ti,ab,kw OR "Infusion, Intravenous":ti,ab,kw OR "Intravenous Infusion":ti,ab,kw OR "Intravenous Drip":ti,ab,kw OR "Drip, Intravenous":ti,ab,kw OR "Drip Infusions":ti,ab,kw OR "Drip Infusion":ti,ab,kw OR "Infusion, Drip":ti,ab,kw OR "Infusions, Drip":ti,ab,kw OR "intravenous":ti,ab,kw OR "intravenous lipid emulsion":ti,ab,kw	85,606
#20	[mh "Infusions, Parenteral"]	12,384
#21	"Infusions, Parenteral":ti,ab,kw OR "Parenteral Infusions":ti,ab,kw OR "Infusion, Parenteral":ti,ab,kw OR "Parenteral Infusion":ti,ab,kw OR "Intra-Abdominal Infusions":ti,ab,kw OR "Infusion, Intra-Abdominal":ti,ab,kw OR "Infusions, Intra-Abdominal":ti,ab,kw OR "Intra Abdominal Infusions":ti,ab,kw OR "Intra-Abdominal Infusion":ti,ab,kw OR "Peritoneal Infusions":ti,ab,kw OR "Infusion, Peritoneal":ti,ab,kw OR "Infusions, Peritoneal":ti,ab,kw OR "Peritoneal Infusion":ti,ab,kw OR "Intraperitoneal Infusions":ti,ab,kw OR "Infusion, Intraperitoneal":ti,ab,kw OR "Infusions, Intraperitoneal":ti,ab,kw OR "Intraperitoneal Infusion":ti,ab,kw OR "parenteral":ti,ab,kw	10,293
#22	[mh "Parenteral Nutrition"]	1,653
#23	"Parenteral Nutrition":ti,ab,kw OR "Nutrition, Parenteral":ti,ab,kw OR "Parenteral Feeding":ti,ab,kw OR "Feeding, Parenteral":ti,ab,kw OR "Feedings, Parenteral":ti,ab,kw OR "Parenteral Feedings":ti,ab,kw OR "Intravenous Feeding":ti,ab,kw OR "Feeding, Intravenous":ti,ab,kw OR "Feedings, Intravenous":ti,ab,kw OR "Intravenous Feedings":ti,ab,kw	4,202
#24 Combine	{OR #14-#23}	151,671
#25 Combine	#13 AND #24	3,493
