# Supplementary figures and images for: Safety and Efficacy of Early High Parenteral Lipid Supplementation in Preterm Infants: A Systematic Review and Meta-Analysis
Source: Nutrients. 2021 May 2;13(5):1535. doi: 10.3390/nu13051535 (PMC8147506; doi:10.3390/nu13051535)

# Highest serum total bilirubin level in hospitalization, mg/dL

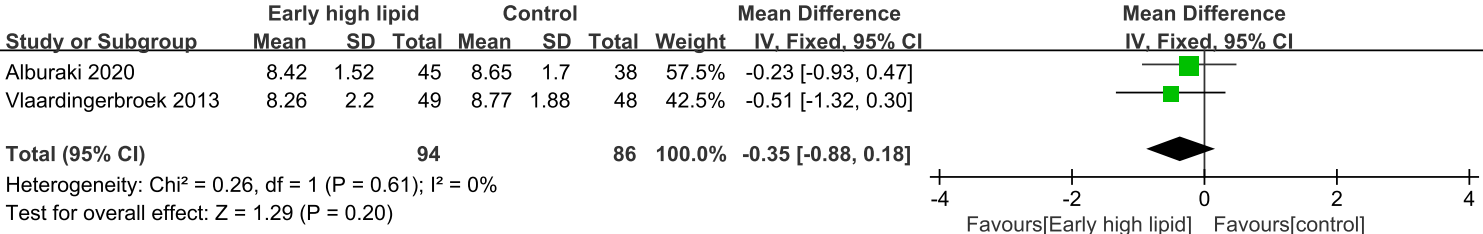

Supplement: Supplementary file 1 [file nutrients-13-01535-s001.zip › supplementary Figure S1.pdf]
